# The effects of restraint stress and orthodontic tooth movements on the intestinal epithelial structure and metabolic function in rats

**DOI:** 10.1371/journal.pone.0319779

**Published:** 2025-02-27

**Authors:** Ye Cheng, Yue Li, Ziqing Fan, Nannan Wang, Min Wang, Yanfen Li, Chao Liu, Huang Li, Fuhua Yan

**Affiliations:** Nanjing Stomatological Hospital, Affiliated Hospital of Medical School, Institute of Stomatology, Nanjing University, Nanjing, China; Hamadan University of Medical Sciences, ISLAMIC REPUBLIC OF IRAN

## Abstract

Chronic stress and orthodontic treatment have been revealed to trigger systemic stress responses in rats. This study aimed to investigate the effects of restraint stress and orthodontic treatment on the intestinal epithelial structure, barrier function, flora, and metabolism in rats. Twenty 8-week-old male Wistar rats were randomly divided into four groups: sham-stressed non-orthodontic (CC), sham-stressed orthodontic (CO), stressed non-orthodontic (SC), and stressed orthodontic (SO). The stress intervention involved subjecting the rats to restraint stress for 21 days, while the orthodontic intervention consisted of maxillary first molar traction from days 8 to 21. Histological and immunohistochemical staining were used to observe the epithelial structure and barrier function of the colon. The intestinal flora and metabolite alterations were investigated by 16S rRNA high-throughput sequencing and untargeted metabolomics sequencing. Colonic epithelial tissue disruption, mucus cells reduction, and a decreased expression of intestinal tight junction proteins were observed in the CO, SC, and SO groups. *Lactobacillus* spp. abundance was significantly lower in the CO group than in the CC group. *Prevotella* spp. abundance was significantly lower in the SC and SO groups than in the CC and CO groups. The differential metabolite enrichment pathways between each inter-group comparison might all be related to amino acid biosynthesis, protein digestion and absorption, and cofactor biosynthesis. Both restraint stress and orthodontic treatment may adversely affect the colonic epithelial structure and barrier function of rats. The intestinal flora structure and types of metabolites were also affected cumulatively.

## 1 Introduction

Orthodontic treatment involves applying orthodontic forces to the teeth through an orthodontic appliance so that the teeth are displaced in the alveolar bone in the direction of the orthodontic forces [1]. With increasing attention being paid to the psychological state of patients, the impact of stress on patients undergoing orthodontic treatment cannot be ignored [2]. There are many types of animal stress: stress to the entire body [3], immune stress triggering immune responses [4], oxidative stress in cells [5], etc. The stress applied in the current study is the systemic non-specific adaptive response that occurs when the body is stimulated by internal and external factors [3]. Long-term restraint treatment for a fixed period per day is a common method of establishing a chronic stress model in rats [6, 7]. In our previous study, restraint stress treatment combined with orthodontic intervention in rats was used to simulate adult orthodontic patients in a chronic stress state. The results showed that orthodontic treatment can lead to changes in body weight, psychological state, and serum corticosterone levels in rats. This was similar to the changes observed under chronic restraint stress.

Chronic stress stimuli are associated with gastrointestinal dysfunction and can lead to irritable bowel syndrome and stress-related gastrointestinal ulcers [8]. The effects of stress on the intestine may be related to the impact on intestinal barrier function, immune function, motility and flora. Stress may lead to an increased expression of corticotropin-releasing hormone, activation of mast cells, increased intestinal epithelial permeability, and intestinal bacterial translocation [9]. Stress affects intestinal motility, including decreasing transmissions in the small intestine [10] and the colon [11]. Furthermore, changes in the intestinal environment can significantly affect intestinal flora structure [12]. The stress-induced decrease in the quantities of *Peptococcaceae* and *Veillonellaceae*can lead to significant reduction in butyrate metabolism, which is essential for maintaining intestinal health [13].

According to our previous study, both chronic restraint stress and orthodontic tooth movements may trigger a systemic stress response in rats [14]. The systemic stress response is at high risk of adversely affecting the intestine. However, the effects of chronic restraint stress combined with orthodontic treatment on the intestine have not been investigated comprehensively. Therefore, the aim of this research was to investigate the effects of chronic stress and orthodontic treatment on the intestine as individual factors and to explore the effects of orthodontic treatment on the intestine when the body is already under chronic stress.

## 2 Materials and methods

### Animal grouping and treatment

Twenty 8-week-old male Wistar rats (200±10 g) [14, 15], SPF grade, were fed with the same diet and water. After 1 day of acclimatization, the rats were randomly divided into four groups: 1) sham-stressed non-orthodontic (CC), 2) sham-stressed orthodontic (CO), 3) stressed non-orthodontic (SC), and 4) stressed orthodontic (SO). To establish the model of adult patients exposed to chronic stress before orthodontic treatment, restraint stress intervention continued from day 0 to day 21, while the orthodontic intervention was conducted from days 8 to 21. All experimental procedures were reviewed and approved by the Animal Ethics Committee of Nanjing Agricultural University (No. PZ2023038).

For the restraint stress intervention, each rat in the SC and SO groups was placed in a sterilized polyethylene glycol terephthalate cylindrical tube (12 cm long, 5 cm outer diameter) with air holes for ventilation. The restraint stress intervention lasted for 2 hours daily from 8:00 AM to 10:00 AM. Rats in the CC and CO groups were captured, briefly held in hand, and then promptly returned to their cages at the same time points as those of the sham stress intervention each day [16].

After anesthetizing the rats in the CO and SO groups with isoflurane (concentration 3%, flow rate 1 L/min), the orthodontic devices were installed in their mouths following the steps detailed previously [15]. The maxillary first molar was pulled mesially with an initial force of 40 g. Orthodontic force was applied from days 8 to 21. The rats in the non-orthodontic intervention groups received the same anesthesia during the same period [14].

### Intestinal histological examination

On day 21, all rats were anesthetized, and then, euthanized by cervical dislocation. The colonic tissues were sampled and fixed in a 4% paraformaldehyde aqueous solution for 48 hours, followed by routine dehydration and paraffin embedding. Paraffin sections of approximately 3-μm thickness and perpendicular to the direction of the intestinal tract were prepared.

Sections of intestines were stained with hematoxylin-eosin (HE) for histomorphology. Structural integrity, intestinal villi, crypts, and other structural and morphological changes, as well as inflammatory cell infiltration, were assessed. Sections were stained with Alcian Blue-Periodic Acid Schiff (AB-PAS) to observe the morphology, number, and distribution of mucous cells. Stained sections were scanned with a digital scanner to collect images.

### Immunohistochemical testing

Colon sections were subjected to immunohistochemical staining. The sections underwent routine antigen retrieval, blocking of endogenous peroxidase, and serum-blocking, followed by the addition of primary and secondary antibodies, 3,3’-Diaminobenzidine (DAB) chromogenic staining, and subsequent counterstaining of the nuclei. This process was used to detect the expression, distribution, and changes of intestinal junction proteins, including occludin, zonula occludens-1 (ZO-1), and claudin-1. The primary antibodies and their dilution ratios were as follows: anti-occludin antibody (Servicebio, GB111401, 1:500), anti-ZO-1 antibody (Servicebio, GB111402, 1:800), and anti-claudin-1 antibody (Servicebio, GB11032, 1:500). Stained sections were scanned with a digital scanner to collect images, and semi-quantitative analysis was conducted on the proportion of staining-positive area within a fixed-size region of the images.

### Intestinal flora and metabolomics testing

After euthanizing the rats on day 21, the cecum segment of the rats was carefully isolated; its contents were extruded, and immediately frozen and stored in a -80°C refrigerator.

#### 16S rRNA sequencing analysis

The NSive COyes classifier was used in QIIME2 software for species annotation at different taxonomic levels against the Silva database. The Chao1 and Observed species indices were used to characterize species richness. Diversity was characterized by the Shannon and Simpson indices. Faith’s phylogenetic diversity index was used to characterize genetic diversity. Community evenness was characterized by Pielou’s evenness index. Good’s coverage index was used to characterize coverage. Rarefaction curves were plotted to assess the sequencing depth of the data. The Jaccard distance of the samples was calculated to generate a sample difference distance matrix. Principal coordinates analysis (PCoA) was used to perform sorting to obtain the PCoA scatter plot. Subsequently, hierarchical clustering analysis was conducted to obtain the dendrogram to characterize the similarity between the samples. Linear discriminant analysis Effect Size (LEfSe) analysis was employed to examine the differences in species composition across the different groups. Significantly differentiated species are presented with linear discriminant analysis scores. A cladogram was used to intuitively present the distribution of marker species across various taxonomic levels in different groups.

#### Metabolomics analysis

Filtered supernatant (20 μL) was aspirated from each assay vial and mixed as a quality control (QC) sample. Differences in QC samples were inspected to assess systematic errors, while also ensuring quality assurance by removing feature peaks with poor reproducibility in QC samples. Initially, the data underwent autoscaling, followed by unsupervised Principal Component Analysis (PCA) to display the original state of the metabolomics data; next, Orthogonal Projections to Latent Structures-Discriminant Analysis (OPLS-DA) between the CC and CO groups, the CC and SC groups, and the CC and SO groups were performed. Differential metabolites between groups were selected based on Variable Importance in Projection (VIP) ≥ 1 and P ≤ 0.05. Subsequently, the exact molecular weight of these metabolites was determined (with a molecular weight error of less than 30 ppm) using the fragment information obtained from tandem mass spectrometry spectra. This information was compared with several databases, including Metlin, Human Metabolome Database, MassBank, LipidMaps, and mzCloud, followed by KEGG pathway enrichment analysis of the differential metabolites.

### Statistical analysis

The experimental data are expressed as mean ± standard deviation (X ± SD), and were statistically analyzed and plotted using Graph Pad Prism 8.0 software, with one-way analysis of variance or Kruskal-Wallis test for comparisons between multiple groups. P < 0.05 was considered statistically significant.

## 3 Results

### Alterations of colonic epithelium structure and the epithelial barrier

**Fig 1:**
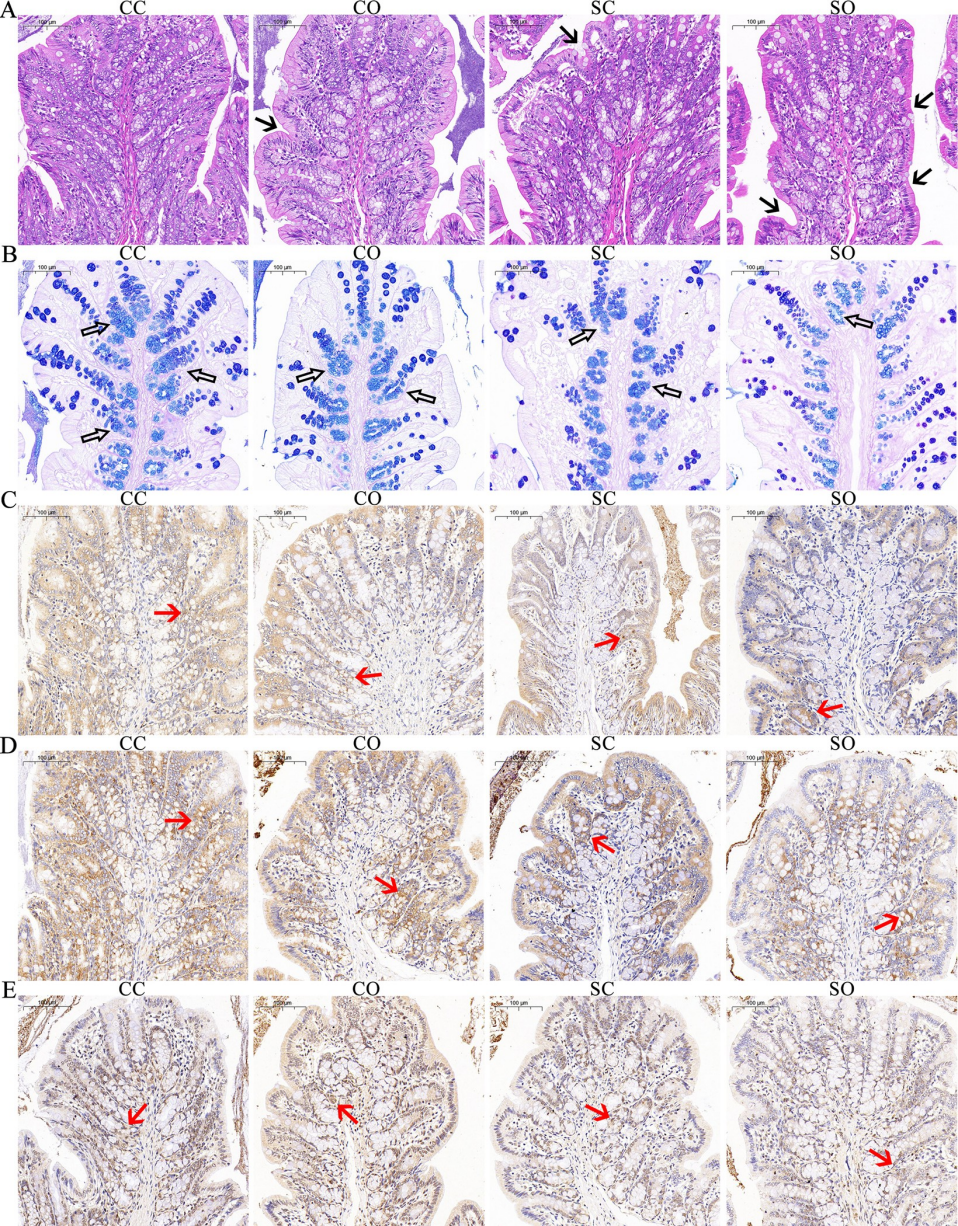
Staining of colonic tissue sections in rats. (A) Hematoxylin-eosin (HE) staining (black arrows: areas of altered epithelial architecture); (B) Alcian Blue-Periodic Acid Schiff (AB-PAS) staining (hollow arrows: mucous cells stained blue); (C) Immunohistochemical staining of occludin; (D) Immunohistochemical staining of zonula occludens-1 (ZO-1); (E) Immunohistochemical staining of claudin-1. (red arrows: positive immunohistochemical staining of occludin, ZO-1, and claudin-1; scale bar: 100 μm).

The colonic epithelium in the CC group was revealed to be intact, and the crypts were regularly arranged. In contrast, the colon tissues in the CO, SC, and SO groups were disordered, and the crypts were unclear and irregularly arranged. The most obvious abnormality was found in the colonic tissue structure of rats in the SO group (Fig 1A). The CC group demonstrated the highest number of goblet cells in the colonic crypts. Conversely, the number of goblet cells in the colonic crypts of rats in the SO group was significantly reduced (Fig 1B).

**Fig 2:**
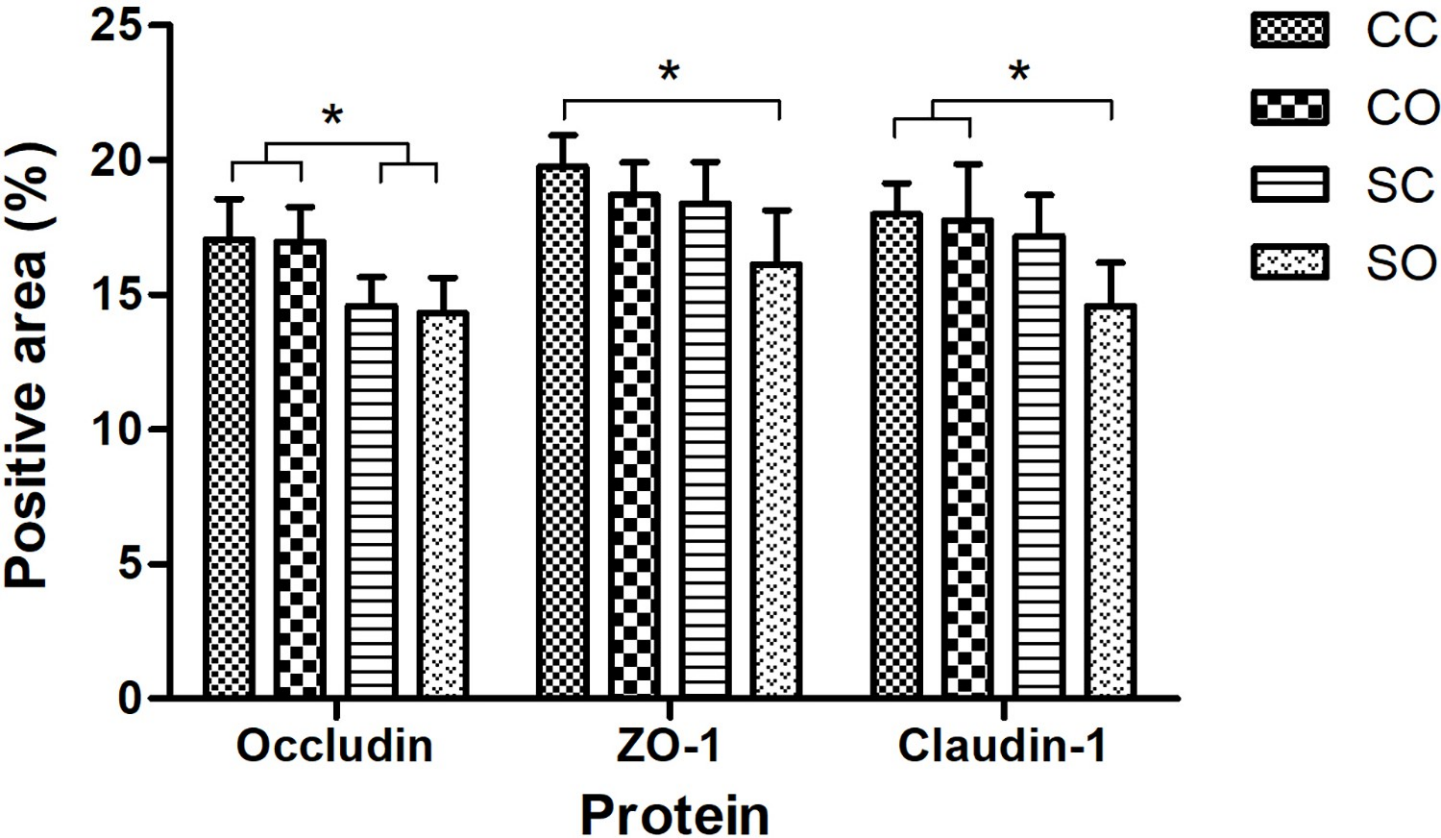
Quantitative results of immunohistochemical staining. (The vertical bar on the tops of the columns indicates standard deviation; *P <0.05).

Positive immunohistochemical staining of occludin, ZO-1, and claudin-1 was observed mostly at the bottom of the crypts of the intestinal wall. There was no significant difference in their expression sites among the four groups (Fig 1C–1E). The expression of occludin was significantly higher in the CC and CO groups than in the SC and SO groups. The expression of ZO-1 was significantly higher in the CC group than in the SO group, and the expression of claudin-1 was significantly lower in the SO group than in the CC and CO groups (Fig 2).

### Impacts on intestinal flora diversity

**Fig 3:**
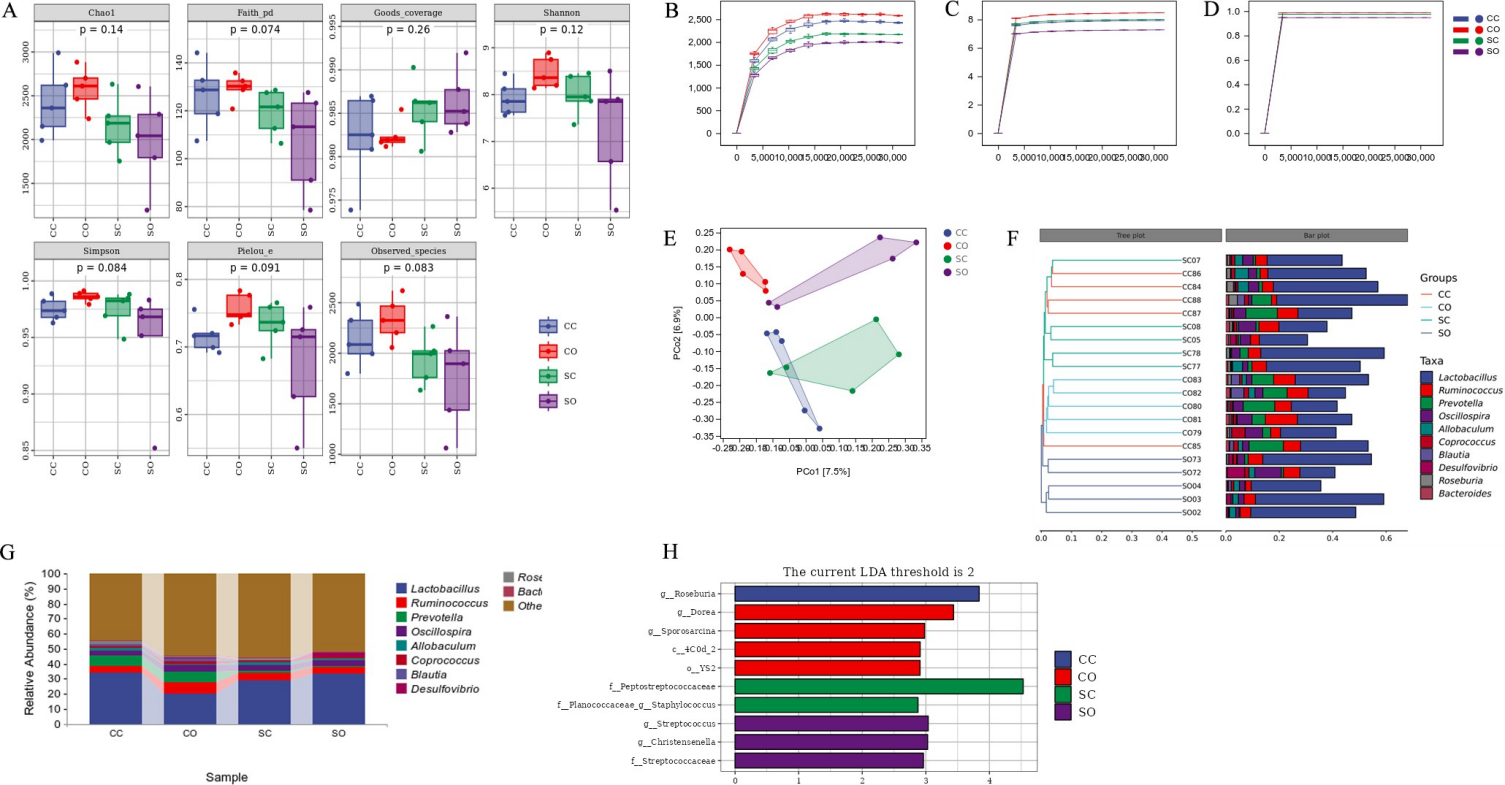
16S rRNA sequence analysis of intestinal microbiota in rats. (A) α diversity index (the median line in the box represents the median, and the upper and lower edges represent the maximum and minimum values.); (B-D) Sparse curves of Chao1, Shannon and Simpson indices (the horizontal coordinate indicates the depth of the flattening, and the vertical coordinate indicates the median value of the diversity index after 10 calculations.); (E) Principal Coordinate Analysis (PCoA) based on the Jaccard distance; (F) Hierarchical cluster analysis based on the Jaccard distance; (G) Taxonomic composition of intestinal microbiota; (H) Linear discriminant analysis Effect Size (LEfSe) analysis of intestinal microbiota (the horizontal coordinate is the logarithmic score of linear discriminant analysis [LDA] for each taxon).

None of the four groups of rats had statistically significant differences in Chao1, Observed species, Shannon, Simpson, Faith’s PD, Pielou’s evenness, and Good’s coverage indices (Fig 3A). The rarefaction curves of Chao1, Shannon, and Simpson indices (Fig 3B–3D) showed that the curves flattened out as the sequencing depth on the X-axis increased. PCoA analysis of the intestinal flora showed no significant difference in intestinal flora composition between the CC and SC groups of rats, but the two groups differed significantly from the CO and SO groups in this metric (Fig 3E). The hierarchical clustering dendrogram revealed that certain differences in intestinal flora composition existed among the four groups (Fig 3F).

### Impacts on intestinal flora species composition

The taxonomic composition of the intestinal flora of the four groups of rats revealed that the dominant bacteria in the cecal contents of all groups were *Lactobacillus* spp., *Ruminococcus* spp., *Prevotella* spp., and *Oscillospira* spp. Among them, the abundance of *Lactobacillus* spp. was significantly higher in the CC group than in the CO group, but the difference was not statistically significant between the SC and SO groups. Moreover, the abundance of *Prevotella* spp. was significantly greater in the CC and CO groups than in the SC and SO groups (Fig 3G). LEfSe analysis comparing the composition of the four groups showed that *Roseburia* was significantly enriched in the CC group, *Dorea* and *Sporosarcina* were significantly enriched in the CO group, *Peptostreptococcaceae* and *Staphylococcus* were significantly enriched in the SC group, and *Streptococcus*, *Christensenella*, and *Streptococcaceae* were significantly enriched in the SO group (Fig 3H).

### Impacts on intestinal metabolomics

**Fig 4:**
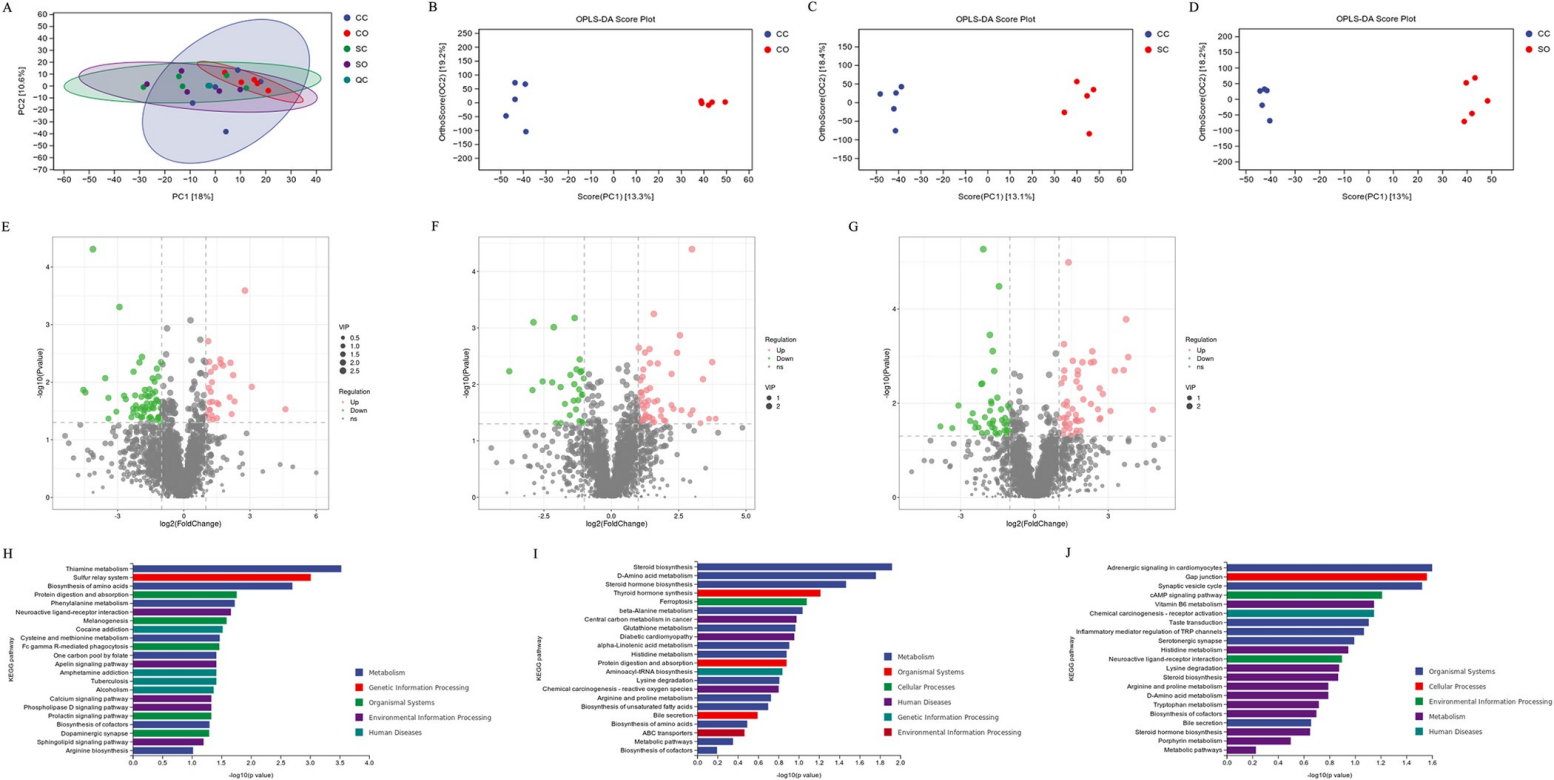
Metabolic analysis of cecal contents. (A) Principal Component Analysis (PCA) of quality control (QC) samples in positive-ion mode; (B) Orthogonal Projections to Latent Structure-Discriminant Analysis (OPLS-DA) of sham-stressed non-orthodontic (CC) vs. sham-stressed orthodontic (CO) in positive-ion mode; (C) OPLS-DA analysis of CC vs. stressed non-orthodontic (SC) in positive-ion mode; (D) OPLS-DA analysis of CC vs. SO in positive-ion mode; (E) Volcano map of CC vs. CO in positive-ion mode; (F) Volcano map of CC vs. SC in positive-ion mode; (G) Volcano map of CC vs. SO in positive-ion mode; (H) KEGG pathway analysis of differential metabolites between CC and CO in positive-ion mode; (I) KEGG pathway analysis of differential metabolites between CC and SC in positive-ion mode; (J) KEGG pathway analysis of differential metabolites between CC and SO in positive-ion mode.

Under both positive and negative ion modes, QC samples were clustered together (Fig 4A). Meanwhile, the proportions of characteristic peaks with the relative SD ≤ 30% were above 80%. The two groups in each pair of OPLS-DA could be completely separated from each other (Fig 4B–4D). The model evaluation parameters (R2Y, Q2) obtained through seven-fold cross-validation are listed in Table 1, where the Q2 values for all three comparisons were greater than 0.3. Based on the VIP values of the first principal component in OPLS-DA and the P-values from the statistical analysis of metabolites between two groups, volcano plots of metabolites were generated for each pair of groups (Fig 4E–4G). The red points in the volcano plots represent up-regulated differential metabolites, and the green points represent down-regulated differential metabolites. Based on comparison with the database, the numbers of differential metabolites between the CC and CO groups, CC and SC groups, and CC and SO groups were determined to be 172, 149, and 152, respectively (Table 2). KEGG pathway analysis identified differential metabolites between groups (Fig 4H–4J). The differential metabolites between the CC and CO groups, and CC and SC groups were enriched in the following metabolic pathways: biosynthesis of amino acids, protein digestion and absorption, and biosynthesis of cofactors. Of them, the only metabolic pathway where the differential metabolites were enriched between the CC and SO groups was the biosynthesis of the cofactor’s pathway. The major enrichment pathways of the differential metabolites between the CC and SO groups included the adrenergic signaling in cardiomyocytes, gap junction, synaptic vesicle cycle, and cAMP signaling pathways (Fig 4H–4J).

**Table 1:** Evaluation parameters of Orthogonal Projections to Latent Structure-Discriminant Analysis (OPLS-DA) in positive ion mode.

Group	R2X(cum)	R2Y(cum)	Q2(cum)	pre	ort
CC_vs_CO	0.325	0.993	0.426	1	1
CC_vs_SC	0.315	0.991	0.366	1	1
CC_vs_SO	0.312	0.996	0.364	1	1

**Table 2:** Different metabolite quantities between groups.

Group	Pos-up	Pos-down	Neg-up	Neg-down	All changed
CC vs CO	39	63	31	39	172
CC vs SC	64	36	31	18	149
CC vs SO	43	48	29	32	152

## 4 Discussion

The intestinal mucosal barrier consists of a mucus layer, intestinal epithelial cells, tight junction proteins, immune cells, and intestinal microorganisms [17, 18]. Of these, the intestinal mucus layer plays a major role in protecting the intestinal tract from mechanical and chemical stimuli and microbial attack; it also helps to maintain the stability of the internal intestinal environment [19]. Histological observations of the rat colon in the present study demonstrated that both stress and orthodontic tooth movements adversely affected the colonic tissue structure of rats. A reduction in goblet cells disrupted the function of the intestinal mucosal barrier [20]. Elevated corticosterone levels maybe the reason for increased mucosal permeability and decreased protein expression in intestine [21]. The similar impact induced by the two interventions might be related to the indistinctive changes in corticosterone levels that they caused [14].

ZO-1, occluding, and claudins are the main components of the tight junctions, involved in maintaining the function of cell polarity and the tight junction barrier [22, 23]. The expression of these tight junction proteins is often used to reflect the barrier function of the intestinal epithelium [24]. Body stress has been shown to affect the integrity of the junctional complex and decrease the expression of tight junction proteins in humans [25]. In this study, stress and orthodontic tooth movements did not affect the position of tight junction protein expression in the rat colon, but affected their expression level. Stress reduced the expression of occludin, while orthodontic tooth movement did not significantly affect occludin expression. Both stress and orthodontic tooth movements reduced the expression of ZO-1. Moreover, a synergistic effect was detected. However, stress and orthodontic tooth movements had a relatively small effect on the expression of claudin-1. A significant reduction in claudin-1 expression only occurred in the presence of both stimuli. Based on existing investigations, the impact of tight junction proteins in this study might be related to the changes in inflammatory factors [26] and gut microbiota [27, 28] caused by stress responses. On the other hand, the main consequence of intestinal barrier disruption is increased enterogenous endotoxin transported of into body circulation, which in return, may lead to endotoxemia, as well as intestinal bacterial translocation [29, 30].

The results showed that stress and orthodontic tooth movements did not significantly affect the internal diversity of the intestinal flora. However, when considering β-diversity, some compositional changes to the flora structure and bacterial species were noted. First, there were significant intergroup differences in the intestinal flora composition of the four groups of rats. Pronounced differences were noted between the CO and SO groups and between the CC and SC groups, suggesting that orthodontic tooth movements led to changes in intestinal flora. Moreover, the changes were stronger with orthodontic tooth movements than with stress. Long-term changes in flora diversity can affect the stability of intestinal microecology, and the corresponding ecological functions [31].

Furthermore, the differential bacterial species of the intestinal flora in each group were analyzed. We found that neither stress nor orthodontic tooth movements affected the types of dominant bacterial species in intestinal flora, but the effect was reflected in the abundance of the dominant bacteria. Orthodontic treatment significantly reduced the abundance of *Lactobacillus* spp. in the two groups of sham-stressed rats, but this effect was not evident in the two groups of stressed rats, suggesting that stress may have masked the effect of orthodontic tooth movements to some extent. Additionally, stress significantly reduced the abundance of *Prevotella* spp., the dominant bacterial genera found in the rat cecum. This finding suggests that the resident flora of the rat intestine was affected by stress and orthodontic tooth movements [32]. Of these, *Lactobacillus* spp. and *Prevotella* spp. are considered beneficial to the intestine [33, 34]. Both decreases in the abundance of *Lactobacillus* spp. (by orthodontic tooth movements) and *Prevotella*spp. (by stress) may lead to an increase in the relative abundance of other genera, especially some harmful microorganisms. The LEfSe analysis of the composition of the flora confirmed this. *Rothia* spp., enriched in the CC group, contributes to the breakdown of indigestible carbohydrates and produces short-chain fatty acids, especially butyric acid, which has anti-inflammatory properties [35]. *Dorea* spp., enriched in the CO group, has similar properties [36]. *Peptostreptococcaceae*, enriched in the SC group, and *Streptococcaceae*, enriched in the SO group, are generally underrepresented in the intestinal flora of healthy rats and are conditionally pathogenic [37–39]. These intestinal flora alterations might be associated with functional disruption to the colonic barrier.

Intestinal microorganisms can influence intestinal function through direct contact with epithelial cells or indirect regulation of metabolite production [40]. In the pathway analysis of intestinal differential metabolites, we compared the metabolic pathways affected by stress and orthodontic interventions when applied individually. It was found that both interventions affected the intestinal biosynthesis of amino acids, protein digestion and absorption, and cofactor biosynthesis. These findings further confirmed the similarity of the effect of restraint stress and orthodontic tooth movements on the intestine. It has been shown that intestinal amino acid metabolic profiles are disturbed in certain disease development and progression and are highly correlated with intestinal flora dysbiosis and metagenome changes [41, 42]. Therefore, the differences in metabolites and pathways in this study may also be related to changes in the intestinal flora because of the interventions. Interestingly, when both stress and orthodontic tooth movements were present, the primary intestinal metabolic pathways affected had almost no overlap with the pathways affected by either alone. These results suggest that a combination of stress and orthodontic tooth movements may not only exert additive effects but may lead to a new mode of action.

According to this study, attention should be paid to the psychological state of patients undergoing orthodontic treatment. When the patient is already in a state of chronic stress, receiving orthodontic treatment may aggravate the existing intestinal discomfort. Methods that relieve patients’ negative emotions and reduce the discomfort during the orthodontic treatment, would help to reduce adverse reactions in the intestinal environment. Furthermore, the intake of beneficial gut bacteria in the form of probiotics containing Lactobacillus spp. may also alleviate the intestinal symptoms of patients.

However, this study has limitations. The sample size of the study was relatively small. The expression of tight junction proteins was investigated using histochemical staining, which is a semi-quantitative analysis and not as accurate as quantitative analysis. In the orthodontic intervention group, the placement and examination of orthodontic devices might have produced potential stress effects other than the orthodontic treatment. The mechanisms of the effects of stress, orthodontic treatment, and a combination of the two interventions on the intestinal structure, flora, and metabolism in rats, have not been explored further. These shortcomings remained to be improved in our further research.

## 5 Conclusion

In conclusion, both restraint stress and orthodontic treatments adversely affected the structure and barrier function of the colonic epithelium in rats, possibly synergistically. Both stress and orthodontic treatment affected the intestinal flora structure and reduced the abundance of beneficial bacteria. Both interventions induced significant intestinal metabolite alterations. These alterations may be related to the biosynthesis of intestinal amino acids, protein digestion and absorption, and biosynthesis of cofactors pathways.
